# Assessing the impact of World Trade Center (WTC) exposures on post-bronchodilator lung function: Insights from WTC survivor population

**DOI:** 10.1371/journal.pone.0344458

**Published:** 2026-03-10

**Authors:** Ziyue Wang, Jiacheng Ge, Yuyan Wang, Katherine Siu, Roberta Goldring, Beno Oppenheimer, Yongzhao Shao, Joan Reibman, Mengling Liu

**Affiliations:** 1 Department of Population Health, NYU Grossman School of Medicine, New York, New York, United States of America; 2 Department of Medicine, NYU Grossman School of Medicine, New York, New York, United States of America; 3 Department of Cardiothoracic Surgery, NYU Grossman School of Medicine, New York, New York, United States of America; Torbat Heydariyeh University of Medical Sciences, IRAN, ISLAMIC REPUBLIC OF

## Abstract

**Objectives:**

To assess the effects of World Trade Center (WTC) exposures, obesity, and smoking on post-bronchodilator (post-BD) lung function in WTC Survivors.

**Methods:**

Data included 5,243 participants enrolled in WTC Environmental Health Center (WTC EHC) program between 2005 and 2022. WTC-related exposures included dust-cloud exposure and occupational/residential roles. Lung function included post-BD spirometry (FEV_1_, FVC) and impulse oscillometry (R_5_, R_20_, AX). Multivariable linear and quantile regressions assessed associations with WTC exposures, BMI, and smoking, adjusting for demographics.

**Results:**

Dust-cloud exposure and Worker status were associated with elevated AX, R_5_, and R_20_, indicating small airway dysfunction. Spirometry showed minimal impact from dust exposure, though Workers had lower FEV₁ and FVC than Residents. Obesity and smoking were consistently linked to poorer lung function, with effect sizes surpassing WTC exposure. No significant interactions were found between BMI and WTC exposures.

**Conclusions:**

WTC exposures are associated with small airway dysfunction, especially in Workers. Obesity and smoking independently worsen lung function, underscoring the importance of both environmental and physiological risk factors in disaster-exposed populations. Post-BD oscillometry adds critical sensitivity in detecting injury to small airways.

## Introduction

In the aftermath of large-scale disasters, especially those involving structural collapse, civilians are often exposed to complex inhalational toxicants, inflicting both acute and chronic injury to the respiratory system. The World Trade Center (WTC) disaster on September 11, 2001, generated an unprecedented mixture of inhalable particulate matter, caustic dust, and combustion byproducts, posing significant long-term health risks to surrounding community members [1–3]. The WTC Environmental Health Center (WTC EHC) is a surveillance and treatment program for community members (Survivors) with WTC exposures and associated medical and/or mental health symptoms [4] and the first to document adverse health effects in community members [5]. Different from the Responder WTC Health Program Centers of Excellence, which are predominantly male and white, the WTC EHC has nearly 50% women, diverse race/ethnicity, socio-economic status (SES), baseline physical conditions, and exposure patterns [5]. Assessing the long-term impacts of the WTC-related exposures on lung function enhances our comprehension of inhalational injury in this diverse urban civilian population.

The long-term surveillance from the WTC EHC has enabled comprehensive assessments of pulmonary function through both spirometry and oscillometry. These complementary modalities are essential for characterizing the heterogeneous respiratory effects of WTC-related exposures. We and others have previously demonstrated that exposure to WTC dust and debris is associated with persistent lower respiratory symptoms, abnormal lung function, and increased risk of developing obstructive airway disease [5–8]. Specifically, spirometry has revealed large airway dysfunction in subsets of Survivors, including fixed airflow obstruction and accelerated lung function decline [9,10]. Oscillometry, the sensitive measure of peripheral airway abnormalities, has revealed small airway dysfunction, including patients with normal spirometry, supporting its role in identifying early or subtle pathological changes [11–13]. Recently, we showed the differences in associations between WTC exposures and pre-bronchodilator (pre-BD) spirometry and oscillometry measures across the wide spectrum distribution of lung function [8].

Most prior investigations have primarily relied on pre-BD measurements, with limited studies of the decline in post-bronchodilator (post-BD) lung function [13,14]. Pre-BD measures may be affected by reversible factors such as acute bronchoconstriction, whereas post-BD testing measures lung function after the administration of a bronchodilator and provides critical insights into the less reversible components of airway obstruction. Post-BD spirometry is currently required for a diagnosis of chronic obstructive pulmonary disease (COPD) [15–17]. Our previous studies showed that a positive bronchodilator response in small airways, as measured by oscillometry, predicted significant improvement in lung function over time [12]. Residual post-BD abnormalities suggested an irreversible component of small airway injury. These findings highlight the importance of studying small airway function, especially the post-BD responses, to better understand physiological changes in WTC-related and other environmental and occupational lung diseases.

Modifiable factors play a critical role in shaping lung function outcomes. Elevated body mass index (BMI) has emerged as an important determinant and is associated with reduced lung volumes and altered respiratory mechanics, which may exacerbate the respiratory effects of toxic inhalational exposures during the WTC disaster [18]. Obesity can also induce systemic inflammation and oxidative stress, compounding airway injury and impairing recovery [19,20]. Smoking is another well-established risk factor that significantly contributes to lung function decline and increases the risk of developing chronic respiratory diseases [21]. Continued smoking after toxic environmental exposure may accelerate lung damage and hinder recovery. A previous study of WTC firefighters showed that BMI posed a greater risk for the development of WTC lung disease compared to smoking [22]. Given the wide variation in BMI and smoking history within the WTC EHC population, consideration of both factors is essential when evaluating respiratory health and crucial for effective lung function management and the development of targeted treatment strategies for WTC Survivors.

To the best of our knowledge, there is limited information on the joint effects of complex WTC exposures, obesity, smoking, and related covariates.We now examine the differences in post-BD lung function in relation to WTC exposures, utilizing various regression models to analyze the impact of these complex exposures on post-BD spirometry and oscillometry measurements. Such an approach is crucial for a deeper understanding of the reversible and irreversible components of lung damage due to inhalational insults. By exploring these dimensions, we aim to contribute to the broader knowledge of long-term pulmonary health in the aftermath of large-scale environmental disasters.

## Methods and materials

### Study design

The WTC EHC enrolls individuals who were exposed to WTC dust/fume and are certified for any WTC-related condition (aerodigestive disorders, acute traumatic injury, cancer, or mental health disorders). Individuals undergo comprehensive physical and mental health evaluations, as well as lung function testing consisting of pre- and post- BD spirometry and oscillometry. Participation required written informed consent, with the study approved by the New York University School of Medicine’s Institutional Review Board (NCT00404898). All research adhered to the ethical standards outlined in the 2013 Helsinki Declaration and its amendments.

### Exposures and covariates

Participants completed a detailed interview-based questionnaire upon enrollment, covering demographic details, exposure details, and health symptoms. This research analyzed data from patients enrolled between August 1, 2005, and December 31, 2022, including updated questionnaire data and available pulmonary measurements. The WTC-related exposures were assessed using two distinct characterizations. The first was a binary acute exposure variable based on the self-reported answer of whether being caught in the dust cloud on September 11, 2001 (dust cloud): ‘Yes’ for caught in the WTC cloud, while ‘No’ for was not. The second was a categorical variable with five groups determined by participants’ reported place of residence and workplace (exposure category): Local Resident, Local Worker, Rescue/Recovery, Clean-up worker, and Other. Detailed description can be found in previous studies [8,23]. Baseline covariates for analysis included age at the initial interview, body mass index (BMI, kg/m^2^) categories (Less than 18.5-Underweight, 18.5 to less than 25-Normal, 25 to less than 30-Overweight, and 30 or greater-Obesity), sex (Female, Male), race/ethnicity (Hispanic, non-Hispanic white, non-Hispanic black, and non-Hispanic other), family income level (Less than or equal to $15,000/year, $15,001 - $30,000/year, and More than $30,000/year, Don’t know/Refused), education level (Equal or less than high school, More than high school), insurance status (No, Yes), and ever smoking status (No, Yes). We also included a categorical variable denoting 5-year intervals since the start of EHC (2005–2009, 2010–2014, 2015–2019, 2010–2024) to account for enrollment cohort effects.

### Lung function measurements

Studies of pulmonary function were conducted at the Andre Cournand Pulmonary Laboratory at Bellevue Hospital. Spirometry was performed utilizing a Vmax spirometer (Vyaire Medical, Irvine, CA). Testing was conducted before and after bronchodilator administration in accordance with the American Thoracic Society (ATS) and European Respiratory Society guidelines. Data for post-BD forced expiratory volume in one second (FEV_1_, unit L) and post-BD forced vital capacity (FVC, unit L) were analyzed using both original raw values and GLI-global %predicted values without race adjustments [24].

Oscillometry was performed with pre- and post-bronchodilation using the Jaeger impulse oscillation system (Vyaire Medical, Irvine, CA) and in line with established guidelines which have previously been reported [24,25]. Impulse oscillometry was performed during tidal breathing with firm support of the cheeks. Trials were included only if tidal breathing and end-expiratory volume were stable and coherence exceeded 0.85 at oscillation frequencies ≥10 Hz. Reproducibility across trials was required, with variability <10%. The specific oscillometry measurements used in this study were post-BD resistance at 5 Hz (R_5_, unit cmH₂O·s/L), post-BD resistance at 20 Hz (R_20_, unit cmH₂O·s/L), post-BD frequency dependence of resistance calculated as the difference between R5 and R20 (R_5-20_, unit cmH₂O·s/L), and post-BD the area above the reactance curve from 5 Hz to the resonant frequency (AX, unit cmH₂O/L). Both original raw values and z-scores based on the predictive equation in [26] were provided. Only data from trials with constant tidal volume and end-expiratory volume were analyzed to improve the data quality.

### Statistical analyses

Summary statistics were presented for lung function outcomes, WTC exposures, and patient demographic characteristics. Categorical variables were summarized with counts and proportions, normal continuous variables with mean and standard deviation (SD), and non-normal continuous variables with median and interquartile range (IQR). Comparisons across the WTC dust-cloud groups were conducted using the Wilcoxon rank-sum test for continuous variables and Chi-square test for categorical variables. We considered multiple modeling approaches to examine the associations between various WTC exposure characterizations and post-BD lung function. The first approach conducted multivariable linear regressions to evaluate the differences in the average spirometry measures and the log-transformed oscillometry measures across the exposure groups. Furthermore, to comprehensively assess the impact of WTC exposures on a wide spectrum of the distribution of lung function metrics, we applied quantile regressions on original scale of both spirometry and oscillometry measures, which estimated variations in conditional quantiles (25^th^, 50^th^, and 75^th^ percentiles) among the different exposure groups. All the analyses were adjusted for baseline covariates, including age, BMI categories, sex, race/ethnicity, family income level, education level, insurance status, smoking status, and cohort interval. Height was adjusted further in spirometry analyses. In regression modeling, the categorical exposure category was analyzed with three levels of Worker, Resident and Others, where Rescue or Recovery and Clean-up workers were combined into Others due to the smaller sample size, and BMI was analyzed with three levels of Obese, Overweight, and Underweight/Normal [27]. We also removed data with ‘No Answer’ in the covariates due to small sample size to ensure statistical power and stability of the regression model. As a sensitivity analysis to further investigate the impacts of the complex WTC exposures on lung function across BMI profiles, we tested for interaction effects between WTC exposures and BMI. Statistically significant was declared if p < 0.05. All statistical analyses were performed in R (version 4.3.1).

## Results

### Study population and inclusion criteria

**Fig 1:**
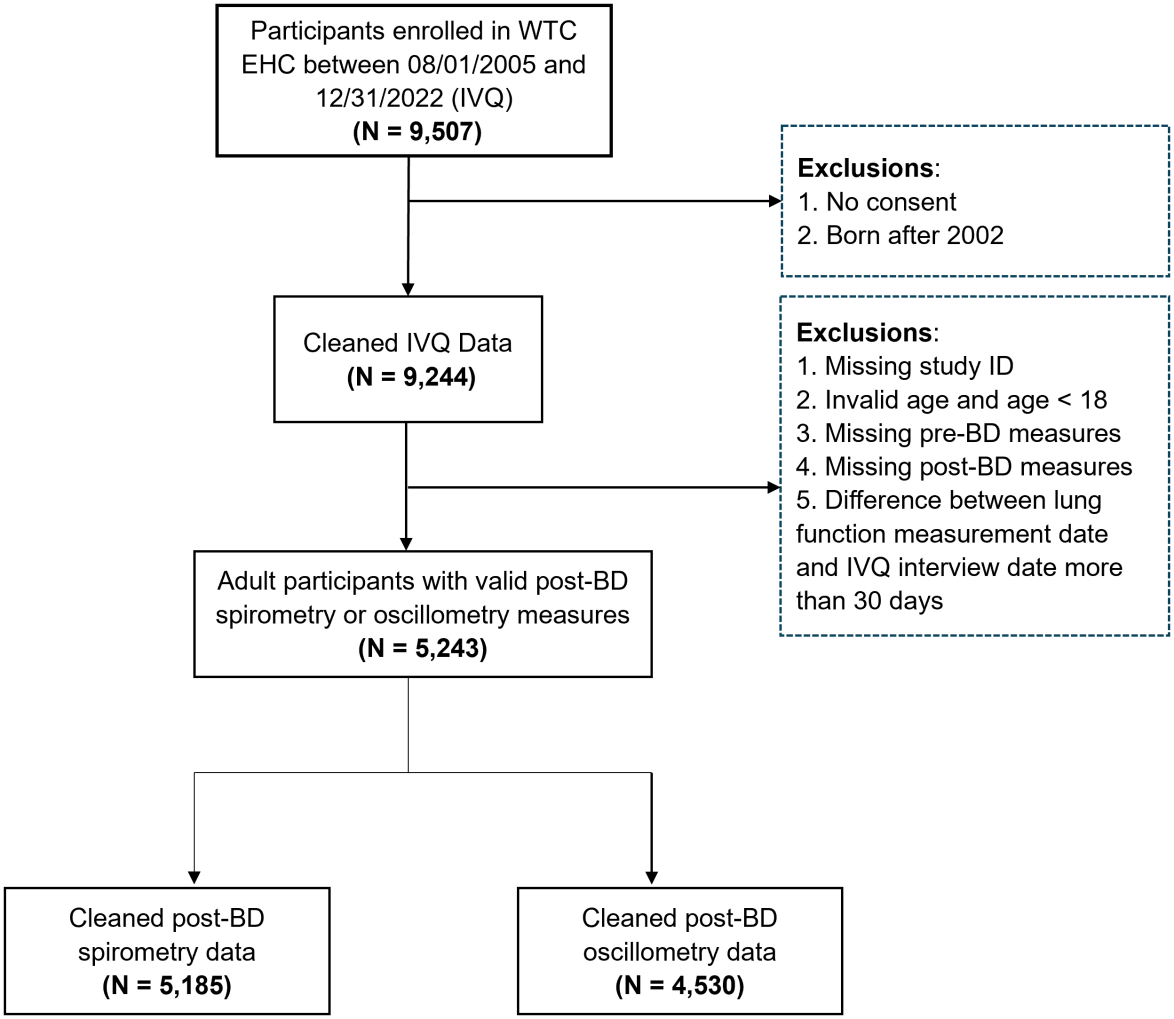
Flowchart of data pre-processing. Participants were enrolled and measured with lung functions. IVQ: initial visit questionnaire; post-BD: post-bronchodilator.

The inclusion and exclusion process to identify subjects for the final analysis is illustrated in Fig 1. Initially, 9507 participants were enrolled from August 1, 2005 until December 31, 2022, into the WTC EHC. Individuals were excluded if: born after 2002, missing consent, missing or erroneous data including study ID, age, pre-BD measures, post-BD measures, or misalignment of lung function measurement date from the enrollment date. A total of 5243 participants with valid post-BD measures were retained for further analysis, including 5185 participants with post-BD spirometry and 4530 with post-BD oscillometry, respectively.

### Summary of patient characteristics, spirometry, and oscillometry measurements

Table 1 shows the demographic characteristics of 5243 participants, of which, 2884 (55.0%) were exposed to the dust cloud. Almost half of the participants were women (2608, 49.7%). A majority (2453, 46.8%) were Non-Hispanic White, followed by Hispanic (1145, 21.8%), Black (1137, 21.7%), and Other (507, 9.7%). Many were overweight (1858, 35.6%) or obese (1897, 36.3%). The baseline patient characteristics of age, race/ethnicity, and insurance status were significantly different between dust-cloud exposure groups. Most of the participants were classified as Worker (3307, 63.3%), with fewer Resident (1094, 20.9%), and very few were Rescue or Recovery (110, 2.1%) or Clean-up workers (255, 4.9%). The classifications differed between dust cloud groups, with more Workers reporting dust cloud exposure (p < 0.001).

**Table 1:** Summary characteristics and post-BD lung function by dust cloud group.

		Dust cloud			
Variable	Overall	Yes	No	Missing	P^1^
n	5243	2884	2339	20	
**Exposure category, n (%)**					<0.001
Worker	3307 (63.3)	2043 (71.1)	1256 (53.9)	8 (40.0)	
Resident	1094 (20.9)	541 (18.8)	551 (23.7)	2 (10.0)	
Clean-Up Worker	255 (4.9)	53 (1.8)	196 (8.4)	6 (30.0)	
Rescue/Recovery	110 (2.1)	37 (1.3)	73 (3.1)	0 (0.0)	
Other	457 (8.7)	200 (7.0)	253 (10.9)	4 (20.0)	
**Cohort** ^**2**^ **, n (%)**					<0.001
2005-2009	1555 (29.7)	784 (27.2)	754 (32.2)	17 (85.0)	
2010-2014	1421 (27.1)	824 (28.6)	596 (25.5)	1 (5.0)	
2015-2019	2099 (40.0)	1189 (41.2)	908 (38.8)	2 (10.0)	
2020-2024	168 (3.2)	87 (3.0)	81 (3.5)	0 (0.0)	
**Age, years, mean (SD)**	55(12)	55.05 (11.84)	54.16 (12.43)	48 (13)	0.009
**Height, meters, mean (SD)**	1.68 (0.10)	1.68 (0.10)	1.68 (0.10)	1.62 (0.11)	0.314
**Sex, n (%)**					0.997
Women	2608 (49.7)	1434 (49.7)	1162 (49.7)	12 (60.0)	
Men	2635 (50.3)	1450 (50.3)	1177 (50.3)	8 (40.0)	
**BMI, n (%)**					0.067
Underweight	48 (0.9)	25 (0.9)	23 (1.0)	0 (0.0)	
Normal	1420 (27.2)	742 (25.8)	674 (29.0)	4 (20.0)	
Overweight	1858 (35.6)	1040 (36.1)	811 (34.9)	7 (35.0)	
Obese	1897 (36.3)	1071 (37.2)	817 (35.1)	9 (45.0)	
**Race/Ethnicity, n (%)**					0.001
Hispanic	1145 (21.8)	576 (20.0)	557 (23.8)	12 (60.0)	
Non-Hispanic Other	507 (9.7)	269 (9.3)	235 (10.0)	3 (15.0)	
Non-Hispanic White	2453 (46.8)	1366 (47.4)	1083 (46.3)	4 (20.0)	
Non-Hispanic Black	1137 (21.7)	672 (23.3)	464 (19.8)	1 (5.0)	
No Answer for Either Race or Ethnicity	1 (0.0)	1 (0.0)	0 (0.0)	0 (0.0)	
**Individual income, n (%)**					0.671
Less than or equal to $15,000/year	1531 (29.2)	855 (29.6)	670 (28.6)	6 (30.0)	
$15,001 - $30,000/year	742 (14.2)	403 (14.0)	338 (14.5)	1 (5.0)	
More than $30,000/year	2803 (53.5)	1533 (53.2)	1265 (54.1)	5 (25.0)	
Do not know/Refused	167 (3.2)	93 (3.2)	66 (2.8)	8 (40.0)	
**Ever Smoking, n (%)**					0.535
No	3130 (59.7)	1743 (60.4)	1379 (59.0)	8 (40.0)	
Yes	2100 (40.1)	1136 (39.4)	955 (40.8)	9 (45.0)	
No Answer	13 (0.2)	5 (0.2)	5 (0.2)	3 (15.0)	
**Insurance, n (%)**					0.004
Insured	4595 (87.6)	2569 (89.1)	2015 (86.1)	11 (55.0)	
Uninsured	616 (11.7)	303 (10.5)	308 (13.2)	5 (25.0)	
No Answer	32 (0.6)	12 (0.4)	16 (0.7)	4 (20.0)	
**Education, n (%)**					0.078
Equal or less than high school	1259 (24.0)	655 (22.7)	593 (25.4)	11 (55.0)	
More than high school	3981 (75.9)	2227 (77.2)	1745 (74.6)	9 (45.0)	
No Answer/Never Record/Refused	3 (0.1)	2 (0.1)	1 (0.0)	0 (0.0)	
**Spirometry**					
n	5185	2858	2308	19	
**FEV**_**1**_ **, L, mean (SD)**	2.82 (0.86)	2.81 (0.86)	2.84 (0.86)	2.74 (0.89)	0.182
**FVC, L, mean (SD)**	3.60 (1.05)	3.58 (1.06)	3.63 (1.03)	3.41 (1.06)	0.125
**FEV**_**1**_ **/FVC, %, mean (SD)**	78.40 (8.58)	78.50 (8.41)	78.27 (8.78)	80.87 (9.58)	0.348
**%Predicted FEV**_**1**_^**3**^ **, mean (SD)**	95.08 (18.72)	94.66 (18.71)	95.60 (18.73)	95.74 (16.61)	0.075
**%Predicted FVC** ^**3**^ **, mean (SD)**	97.28 (17.87)	96.61 (17.90)	98.11 (17.81)	97.55 (16.32)	0.003
**Oscillometry**					
n	4530	2523	1993	14	
**R**_**5**_ **, cmH₂O·s/L, median [IQR]**	3.95 [3.11, 5.05]	4.01 [3.14, 5.14]	3.89 [3.04, 4.92]	4.44 [3.23, 5.26]	<0.001
**R**_**20**_ **, cmH₂O·s/L, median [IQR]**	3.11 [2.51, 3.84]	3.14 [2.54, 3.88]	3.06 [2.49, 3.80]	3.28 [2.68, 4.01]	0.015
**R**_**5-20**_ **, cmH₂O·s/L, median [IQR]**	0.79 [0.42, 1.32]	0.82 [0.44, 1.35]	0.75 [0.39, 1.26]	0.74 [0.27, 1.62]	<0.001
**AX, cmH₂O/L, median [IQR]**	4.84 [2.35, 9.85]	5.12 [2.54, 10.30]	4.41 [2.21, 9.20]	3.50 [2.65, 7.98]	<0.001
**R**_**5**_ **(z-score)**^**4**^**, median [IQR]**	1.00 [0.04, 2.28]	1.06 [0.10, 2.34]	0.94 [−0.04, 2.18]	1.40 [0.20, 2.59]	0.001
**R**_**20**_ **(z-score)**^**4**^**, median [IQR]**	0.87 [−0.06, 2.03]	0.92 [0.00, 2.08]	0.82 [−0.12, 1.97]	1.01 [0.36, 3.09]	0.031
**R**_**5-20**_ **(z-score)**^**4**^**, median [IQR]**	0.49 [−0.35, 1.81]	0.56 [−0.30, 1.89]	0.37 [−0.42, 1.66]	0.24 [−0.27, 2.61]	<0.001
**AX (z-score)** ^**4**^ **, median [IQR]**	−0.07 [−1.07, 0.95]	−0.01 [−0.97, 1.02]	−0.18 [−1.16, 0.86]	−0.48 [−0.92, 0.18]	<0.001

^1^Statistical tests for comparisons between WTC dust-cloud exposure of “Yes” vs. “No” groups. Wilcoxon rank-sum test was used for continuous variables and the Chi-square test for categorical variables.

^2^5-year intervals since enrollment.

^3^GLI-global %predicted values without race adjustments.

^4^z-score = (Observed value–Predicted value)/RMSE. Predicted values were calculated based on equations from Gochicoa-Rangel et al. [26], where age, sex, height, and BMI were adjusted. RMSE: root mean square error.

Summary statistics of post-BD spirometry and oscillometry measurements in the overall population and by dust cloud exposure are also included in Table 1. Although the average % predicted FVC was within normal for both WTC dust cloud exposed and unexposed, there was a small and statistically significant decrease (p = 0.003) in those with WTC dust cloud exposure. All oscillometry measures of R_5_, R_20_, R_5-20_, and AX were elevated, consistent with larger abnormalities in the dust-cloud exposed group.

### Association between WTC exposure and spirometry measurements

**Fig 2:**
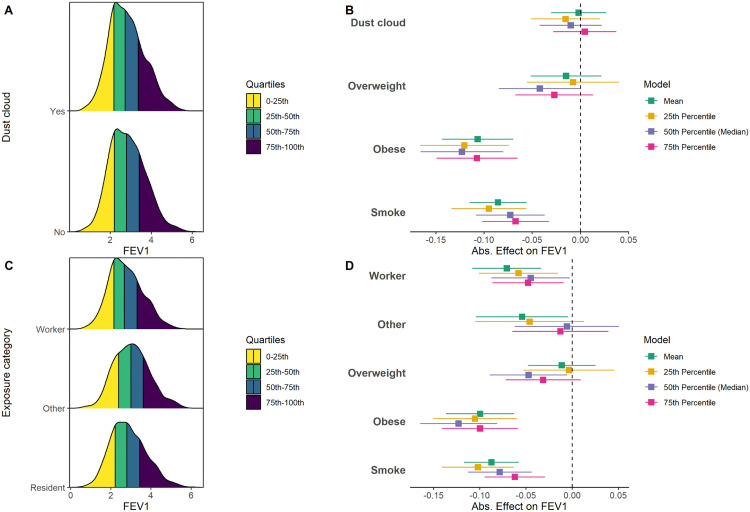
Distributions of FEV₁ and corresponding additive effect estimates from multivariate regression models for dust cloud (A-B) and occupational exposure category (C-D). **A.** Overlaid density plots of FEV₁ for patients caught in dust cloud (top) vs. not caught in dust cloud (bottom). The colored bands represent the four quartile ranges of FEV₁: 0–25th percentile (yellow), 25th–50th (green), 50th–75th (teal), and 75th–100th (navy). **B.** Forest plot of additive effect estimates (with 95% CI) on FEV₁ for evaluating the WTC dust cloud from linear regression (green) and three quantile regressions at the 25th (yellow), 50th (purple), and 75th (pink) percentiles. Normal/underweight is the reference level for BMI. No is the reference level for smoking. X-axis is the absolute effect estimate, and y-axis denotes variables of interests. The vertical dashed line at zero denotes a null effect. **C.** Overlaid density plots of FEV₁ by exposure category (top = Worker; middle = Other; bottom = Resident), with quartile shading same as in **(A)**. **D.** Forest plot of additive effect estimates (with 95% CI) on FEV₁ for evaluating the exposure category from the same setting of regression models as in **(B)**. Reference group is Resident for exposure category. FEV₁: forced expiratory volume in one second.

We used spirometry outcomes in their original scale and thus interpreted the results as the additive change in the mean (linear regression) or in quantile values (quantile regression) when comparing to the reference group. Fig 2 presents the results of post-BD FEV_1_ by WTC exposures along with the estimated additive effect differences on FEV_1_ from both multivariable linear regression and quantile regressions (25^th^, 50^th^, and 75^th^ percentiles), respectively. As shown in Fig 2A, the distribution of FEV_1_ was similar in patients who were caught in dust cloud to those who were not caught in dust cloud at all quantiles. These findings were consistent with the results from both linear and quantile regression when controlling all covariates, where the effect of WTC dust-cloud was not significant in terms of change in post-BD FEV_1_ (Fig 2B).

When comparing the exposure categories (Fig 2C, 2D), the distribution of FEV_1_ in the Workers was worse than the Residents at the mean and all quantiles (Fig 2C). As shown in Fig 2D, the mean of FEV_1_ was significantly reduced in Workers compared to Residents (−0.07, 95% confidence interval [CI]: −0.11, −0.03) after adjusting for other factors, and such significant effects were consistent across the 25^th^ (−0.06, 95%CI: −0.10, −0.02), 50^th^ (−0.04, 95%CI: −0.09, 0.00), and 75^th^ percentiles (−0.05, 95%CI: −0.09, −0.01). The Other group showed a significant reduction of FEV_1_ in mean from linear regression (−0.05, 95%CI: −0.10, 0.00), while no significant effect was identified from quantile regression (Fig 2D). Detailed numerical results are provided in Tables A and B in S1 File.

We also identified significant effects of BMI and ever smoking on the distribution of post-BD FEV_1_. Importantly, the Obese group consistently showed a significant reduction of FEV_1_ at mean and all quantiles compared to those with Normal/underweight BMI. The Overweight group did not consistently show lower FEV_1_ across distribution (Fig 2B, 2D). The effect of smoking on FEV_1_ was significant in both models (Fig 2B, 2D). Overall, obesity and smoking history showed greater decrements of FEV_1_ than WTC-related exposures of either dust-cloud exposure or work-resident exposure categories (Fig 2B, 2D). The interaction between WTC exposures and BMI on FEV_1_ was not significant (dust cloud: p = 0.86; exposure category: p = 0.56), indicating the effect of WTC exposures on spirometry measures was similar across all BMI levels.

Similar effects of WTC-related exposures were observed for FVC, where no significant effect was noted for dust-cloud exposure from either linear or quantile models, while Workers exhibited significantly reduced FVC compared to Residents at the mean, 25^th^ and 50^th^ percentiles (S1 Fig). Higher BMI groups had statistically significantly reduced FVC, particularly, obese patients had the lowest FVC values (S1 Fig). Unlike FEV_1_, smoking was not significantly associated with FVC (S1 Fig). These findings suggested that obesity amplified the most adverse impact on spirometry measures, even more than smoking, and that the Worker group showed a greater reduction in large airway measures, while WTC dust-cloud exposure did not show such an association.

### Association between WTC exposure and oscillometry measurements

**Fig 3:**
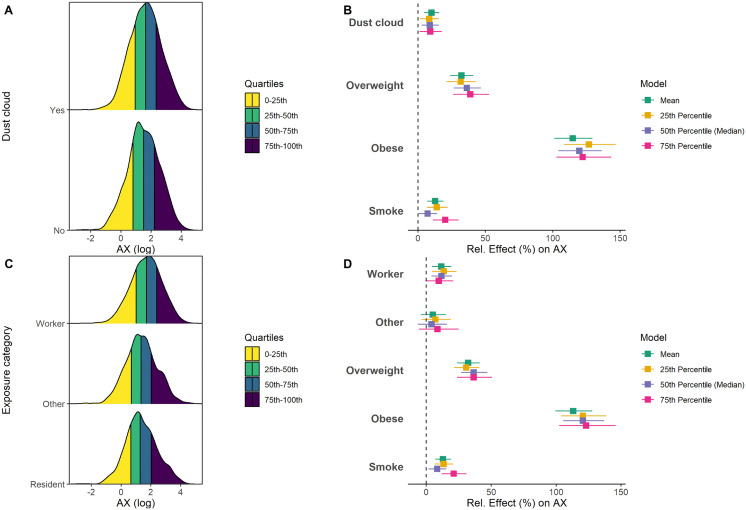
Distributions of log-AX and corresponding relative effect estimates from multivariate regression models for dust cloud (A-B) and occupational exposure category (C-D). **A.** Overlaid density plots of AX on log-scale for patients caught in dust cloud (top) vs. not caught in dust cloud (bottom). The colored bands represent the four quartile ranges of AX: 0–25th percentile (yellow), 25th–50th (green), 50th–75th (teal), and 75th–100th (navy). **B.** Forest plot of relative (%) effect estimates (with 95% CI) on AX for evaluating the WTC dust cloud from linear regression (green) and three quantile regressions at the 25^th^ (yellow), 50^th^ (purple), and 75^th^ (pink) percentiles. Normal/underweight is the reference level for BMI. No is the reference level for smoking. X-axis is the relative (%) effect estimate, and y-axis denotes variables with interests. The vertical dashed line at zero denotes a null effect. **C.** Overlaid density plots of AX on log-scale by exposure category (top = Worker; middle = Other; bottom = Resident), with quartile shading same as in (A). **D.** Forest plot of relative (%) effect estimates (with 95% CI) on AX for evaluating the exposure category from the same setting of regression models as in (B). Reference group is Resident for exposure category. AX: the area above the reactance curve from 5 Hz to the resonant frequency.

Due to the right-skewed distributions of oscillometry measures, log-transformation was applied, and the results were interpreted as the relative change % ((exp(β)−1)×100%) [28] in the geometric mean (linear regression) or in quantile values (quantile regression) of the outcomes when comparing to the reference group. Fig 3 represents the distribution of log-AX by WTC exposures along with estimated relative effect differences on AX. Unlike spirometry measures, we found that overall distributions of AX shifted between patients who were caught in dust cloud versus not caught, where the dust-cloud group had larger AX values at all quantiles (Fig 3A). These findings indicated worse lung function in the dust-cloud exposed group, and the effects of WTC dust-cloud on AX were statistically significant. Specifically, there was a significant increase of AX by WTC dust-cloud at geometric mean level (10.09%, 95%CI: 4.62%, 15.84%), as well as at 25^th^ (8.40%, 95%CI: 1.47%, 15.81%), 50^th^ (8.91%, 95%CI: 2.67%, 15.52%), and 75^th^ (9.20%, 95%CI: 1.33%, 17.67%) percentiles levels (Fig 3B).

When evaluating the groups by exposure category, higher AX values were consistently identified in the Worker Group at all quantiles when comparing to the Resident Group (Fig 3C), with a statistically significant effect of Workers on the geometric mean of AX (11.55%, 95%CI: 4.40%, 19.20%; Fig 3D) at 25^th^ (13.49%, 95%CI: 4.44%, 23.32%) and 50^th^ (11.64%, 95%CI: 3.97%, 19.87%) percentiles (Fig 3D). In contrast, the Other Group did not show a significant elevation on AX from either linear or quantile models (Fig 3D). Detailed results are provided in Tables C and D in S1 File.

BMI was also demonstrated to influence oscillometry measures. In particular, we identified increased AX among both overweight and obese groups compared to normal/underweight BMI patients. The obesity group had the largest effect on AX (Fig 3B, 3D). Additionally, smoking was statistically significantly associated with increasing AX at geometric mean and all quantiles (Fig 3B, 3D). We again did not observe any significant interaction effect between WTC exposures and BMI on AX (dust cloud: p = 0.80; exposure category: p = 0.77).

For other oscillometry measures, we observed that there was a significant worsening of R_5_ and R_20_ in people who were caught in dust cloud, but not with Worker group (S2 and S3 Figs). Again, BMI was significantly associated with higher R_5_ and R_20_ at all quantiles (S2 and S3 Figs). The effect of smoking was more noticeable for R_5_ than R_20_, with significance for R_20_ only identified at 75^th^ percentiles (S2 and S3 Figs). Therefore, our results indicated that severe acute and chronic exposures had the most significant effect on small airway function in patients with relatively normal lung function. Also, BMI and smoking both shifted the entire distribution of small airway lung function towards the worse direction, where the largest impact appeared in obese patients.

## Discussion

We analyzed data from the WTC EHC Survivor population to examine the effects of WTC exposures on post-BD lung function in the diverse civilian population. Our findings demonstrated that exposure to the WTC dust was significantly associated with post-BD small airway dysfunction (elevated AX, R_5_, and R_20_), but not with large airway impairment measured by spirometry. The occupational exposure category (Workers), however, showed significant associations with both small and large airway function, with local Workers experiencing greater reductions in FEV_1_ and increased AX compared to Residents although average values remained within normal limits. Importantly, modifiable factors, particularly BMI and smoking status, had substantial and often greater effects on lung function than WTC exposures, with obesity demonstrating the most pronounced adverse impact on respiratory measurements across both testing modalities.

The use of post-BD measurements in this study provides critical insights into the irreversible components of airway dysfunction following WTC exposure. Post-BD testing, which measures lung function after bronchodilator administration, is particularly valuable for assessing pathological progression, such as airway remodeling, rather than reversible factors like acute bronchoconstriction [29,30]. Our results revealed that oscillometry measures (AX, R_5_, and R_20_) were more sensitive than spirometry in detecting WTC-related respiratory abnormalities, especially in those with dust cloud exposure. This finding aligns with previous studies demonstrating oscillometry's enhanced ability to detect peripheral airway abnormalities, even in patients with normal spirometry [31–33]. In addition, the wide spread of spirometric and oscillometric values reinforces the need to model the full spectrum of lung function outcomes when assessing environmental impacts. Using both quantile regression and linear regression, we found a significant elevation in AX among WTC‑exposed populations. The elevation was identified not only at the geometric mean level but also at lower quantiles, which represent the ostensibly healthier population. Since AX reflects small‑airway reactance and elastance elevated AX after bronchodilation suggests persistent small‑airway remodeling that remains unresponsive to bronchodilation. This finding is particularly concerning as small airway dysfunction may represent early or subtle pathological changes that precede larger airway abnormalities detectable by conventional spirometry [34,35]. Furthermore, our earlier longitudinal research indicated that residual post-BD abnormalities at baseline signaled an irreversible component of small airway injury [6], which appears to be confirmed in this larger cohort analysis. These results underscore the importance of including oscillometry, including post-BD measurements, in clinical assessments of disaster-related respiratory health, as it captures physiological alterations that may be missed by spirometry alone. The differential impact of exposure types on small versus large airways also suggests distinct pathophysiological mechanisms that warrant further investigation to develop targeted therapy approaches for affected individuals.

Our findings highlight the substantial influence of modifiable individual factors on respiratory function among WTC-exposed community populations. Notably, the effects of obesity and smoking on lung function measures consistently exceeded those of WTC exposures across all assessment modalities. Obese individuals demonstrated significantly reduced FEV_1_ and FVC values and markedly elevated oscillometry measurements (AX, R_5_, and R_20_) compared to those with normal BMI. Similarly, smoking history was associated with significant decrements in FEV_1_ and increased small airway dysfunction, particularly evident in elevated AX values. These results align with emerging evidence from WTC firefighter cohorts, suggesting that BMI is a more significant risk factor for developing lung disease compared to smoking [22]. Our previous studies have also reported an association between WTC exposures and increased BMI [23], and the absence of significant interaction effects between WTC exposures and BMI suggests these factors operate independently and additively to impair respiratory function. While WTC-related exposures cannot be reversed, these modifiable risk factors represent actionable targets for therapeutic strategies. Our findings highlight the need to integrate weight management and smoking cessation into comprehensive care plans for WTC survivors [36].

Our study has several notable strengths. The large sample size, diverse population demographics, and comprehensive post-BD lung function testing within the WTC EHC offer a valuable opportunity to examine the long-term respiratory impacts of complex disaster exposures. Unlike most previous research that primarily relied on pre-BD measurements, our inclusion of post-BD spirometry and oscillometry allows for better differentiation between reversible and irreversible components of airway dysfunction. As far as we know, this study is the first to comprehensively evaluate the associations between WTC exposures, BMI, smoking status, and post-BD lung function using both spirometry and oscillometry within the WTC population. Our findings establish a crucial foundation for understanding how modifiable factors might influence respiratory outcomes in environmentally exposed populations.

However, there are also limitations in this study. Because the study used a self-reported cohort whose participation was based on past diagnosis and possible exposure to WTC dust, it may be prone to both recall and selection bias. We further compared the patient characteristics of the WTC EHC to two other major WTC cohorts among the exposed population, Fire Department of the City of New York (FDNY) firefighters and emergency medical service (EMS) workers, and World Trade Center Health Registry (WTCHR) enrollees. Comparing with FDNY responders (n = 15,646), our WTC EHC cohort was older (mean age 55 years) than FDNY median of 40.6 years among firefighters and 34.9 years among EMS workers [37]. The WTC EHC included 49.7% women, whereas FDNY cohorts are overwhelmingly male (96.7% overall) [37]. The WTC EHC cohort was racially/ethnically diverse (46.8% non-Hispanic White, 21.7% non-Hispanic Black, 21.8% Hispanic), in contrast to FDNY responders who were predominantly White (86.7% overall) [37]. The proportion of ever smoking patients in the WTC EHC was 40.1%, similar to FDNY firefighters (34.4% former and 5% current) and lower than EMS (36.1% former and 13.5% current) [37]. We also noted that 71.9% of WTC EHC participants were overweight/obese, similar as FDNY responders (mean BMI > 25) [38]. For the WTCHR cohort (n = 71,437), there was 59.9% male and 63.0% non-Hispanic White, with ever-smoking of 42% (current 15.8%, former 26.2%) [39]. Overall, the WTC EHC is a diverse community cohort and has similar population characteristics to the WTCHR. The differences in designs and basic characteristics among various WTC cohorts warrant cautions in interpreting results from a specific cohort and generalizing to others. Our results most directly reflect symptomatic, treatment seeking group of the exposed population. However, many WTC‑exposed residents and workers without persistent respiratory symptoms did not enroll into the WTC EHC cohort, and these community members might exhibit different lung function patterns than those we observed here. For example, in a healthy, asymptomatic urban cohort of lifetime nonsmokers enrolled in WTC Health Registry, which was the control-group from a previously completed case–control study of the health effects of exposure to WTC dust, respiratory impedance measured by oscillometry was not meaningfully related to dust exposure magnitude and aligned with device‑specific normative ranges, suggesting that exposure alone did not alter oscillometry measures in asymptomatic individuals [40]. We therefore acknowledge limited generalizability and recommend future population-based studies to assess external validity. Additionally, we recognize that BMI is not a perfect indicator of obesity, since it does not distinguish between different body compositions such as muscle mass, fat mass, or bone mass. However, BMI continues to be widely used in large-scale epidemiological studies and serves as a practical indicator of overall adiposity. Our analysis focused on the raw value of post-BD spirometry measures. Although we adjusted the height and sex in the regression models, it’s worth evaluating the results using the % predicted values in the future. Our cross-sectional analysis of post-BD measurements does not capture long-term changes in lung function or allow for assessment of rate of decline. Due to the wide-time spread of the patient enrollment in the cohort, the impact of the lung function might be changed non-monotonically over time, and a previous study has found magnitude of lung function improvement was closely associated with the BD response observed during the initial visit [13]. Thus, longitudinal analysis should be performed in the future. One of the ongoing works from our group leverages the repeated post-BD measurements to model long-term effect on the lung function trajectories. Longitudinal analysis on post-BD measures will highlight the long-term respiratory consequences of WTC exposure and provide insights for targeted interventions to mitigate further lung function decline in this population. Finally, our study did not consider other occupational exposures or environmental sources of exposure, presenting an area for future investigation. Future research should incorporate repeated measures of both exposures and outcomes to better understand the dynamic relationships between modifiable risk factors and respiratory health in this population.

## Conclusion

Aerodigestive disorder is a certified WTC-related health condition, where WTC survivors with these diseases are covered by federally funded health care. However, the joint effects of WTC complex exposures and modifiable factors are not currently well characterized. Our analysis of post‑BD lung function highlights that WTC exposures significantly impact respiratory health, but modifiable risks, especially obesity and smoking, often exert equal or greater harm on pulmonary function independently. By incorporating oscillometry alongside conventional spirometry in clinical assessments, and targeting weight management and smoking cessation, this study offers a potential and practical route of clinical intervention and multifaceted treatments for curbing respiratory decline, creating valuable opportunities for WTC survivors and others affected by complex environmental exposures to improve quality of life.

## Supporting information

S1 File Supplementary tables of the effect estimate on spirometry and oscillometry measures from linear and quantile regression (25th, 50th, and 75th) models for WTC exposure variables. (DOCX)

S1 Fig Distributions of FVC and corresponding additive effect estimates from multivariate regression models for dust cloud (A-B) and occupational exposure category (C-D). **A.** Overlaid density plots of FVC for patients caught in dust cloud (top) vs. not caught in dust cloud (bottom). The colored bands represent the four quartile ranges of FVC: 0–25th percentile (yellow), 25th–50th (green), 50th–75th (teal), and 75th–100th (navy). **B.** Forest plot of absolute effect estimates (with 95% CI) on FVC for evaluating the WTC dust cloud from linear regression (green) and three quantile regressions at the 25th (yellow), 50th (purple) and 75th (pink) percentiles. Normal/underweight is the reference level for BMI. No is the reference level for smoking. X-axis is the absolute effect estimate, and y-axis denotes variables with interests. The vertical dashed line at zero denotes a null effect. **C.** Overlaid density plots of FVC by exposure category (top = Worker; middle = Other; bottom = Resident), with quartile shading same as in (A). **D.** Forest plot of absolute effect estimates (with 95% CI) on FVC for evaluating the exposure category from the same setting of regression models as in (B). Reference group is Resident for exposure category. FVC: forced vital capacity.
(TIF)

S2 Fig Distributions of log-R5 and corresponding relative effect estimates from multivariate regression models for dust cloud (A-B) and occupational exposure category (C-D). **A.** Overlaid density plots of R5 on log-scale for patients caught in dust cloud (top) vs. not caught in dust cloud (bottom). The colored bands represent the four quartile ranges of R5: 0–25th percentile (yellow), 25th–50th (green), 50th–75th (teal), and 75th–100th (navy). **B.** Forest plot of relative (%) effect estimates (with 95% CI) on R5 for evaluating the WTC dust cloud from linear regression (green) and three quantile regressions at the 25th (yellow), 50th (purple) and 75th (pink) percentiles. Normal/underweight is the reference level for BMI. No is the reference level for smoking. X-axis is the relative (%) effect estimate, and y-axis denotes variables with interests. The vertical dashed line at zero denotes a null effect. **C.** Overlaid density plots of R5 on log-scale by exposure category (top = Worker; middle = Other; bottom = Resident), with quartile shading same as in (A). **D.** Forest plot of relative (%) effect estimates (with 95% CI) on R5 for evaluating the exposure category from the same setting of regression models as in (B). Reference group is Resident for exposure category. R5: total airway resistance to 5 Hz.
(TIF)

S3 Fig Distributions of log-R20 and corresponding relative effect estimates from multivariate regression models for dust cloud (A-B) and occupational exposure category (C-D). **A.** Overlaid density plots of R20 on log-scale for patients caught in dust cloud (top) vs. not caught in dust cloud (bottom). The colored bands represent the four quartile ranges of R20: 0–25th percentile (yellow), 25th–50th (green), 50th–75th (teal), and 75th–100th (navy). **B.** Forest plot of relative (%) effect estimates (with 95% CI) on R20 for evaluating the WTC dust cloud from linear regression (green) and three quantile regressions at the 25th (yellow), 50th (purple) and 75th (pink) percentiles. Normal/underweight is the reference level for BMI. No is the reference level for smoking. X-axis is the relative (%) effect estimate, and y-axis denotes variables with interests. The vertical dashed line at zero denotes a null effect. **C.** Overlaid density plots of R20 on log-scale by exposure category (top = Worker; middle = Other; bottom = Resident), with quartile shading same as in (A). **D.** Forest plot of relative (%) effect estimates (with 95% CI) on R20 for evaluating the exposure category from the same setting of regression models as in (B). Reference group is Resident for exposure category. R20: total airway resistance to 20 Hz.
(TIF)
