# Supplementary material for: Assessing the impact of World Trade Center (WTC) exposures on post-bronchodilator lung function: Insights from WTC survivor population
Source: PLoS One. 2026 Mar 10;21(3):e0344458. doi: 10.1371/journal.pone.0344458 (PMC12974862; doi:10.1371/journal.pone.0344458)
Supplement: S1 File — (DOCX) [file pone.0344458.s001.docx]

| Table A. Summary of absolute effect estimates on FEV1 from linear and quantile regression (25th, 50th, and 75th) models for dust cloud. | | | | | | | | |
| --- | --- | --- | --- | --- | --- | --- | --- | --- |
|  | Linear regression | | Quantile regression at 25th percentile | | Quantile regression at 50th percentile | | Quantile regression at 75th percentile | |
| Coefficient | Estimates | 95% CI | Estimates | 95% CI | Estimates | 95% CI | Estimates | 95% CI |
| Cohort, 2010-2014 | 0.05 | (0.02, 0.09) | 0.03 | (-0.02, 0.08) | 0.08 | (0.03, 0.12) | 0.04 | (-0.00, 0.08) |
| Cohort, 2015-2019 | -0.01 | (-0.05, 0.03) | -0.01 | (-0.06, 0.04) | 0 | (-0.04, 0.04) | -0.02 | (-0.07, 0.02) |
| Cohort, 2020-2024 | 0.07 | (-0.02, 0.16) | 0.03 | (-0.03, 0.08) | 0.08 | (-0.03, 0.19) | 0.02 | (-0.08, 0.11) |
| Dust cloud, Yes | 0 | (-0.03, 0.03) | -0.02 | (-0.05, 0.02) | -0.01 | (-0.04, 0.02) | 0 | (-0.03, 0.04) |
| Age | -0.03 | (-0.03, -0.03) | -0.03 | (-0.03, -0.03) | -0.03 | (-0.03, -0.03) | -0.03 | (-0.03, -0.03) |
| Height | 3.25 | (3.05, 3.45) | 2.88 | (2.63, 3.13) | 3.22 | (2.99, 3.45) | 3.38 | (3.15, 3.61) |
| Sex, Female | -0.45 | (-0.49, -0.41) | -0.41 | (-0.46, -0.36) | -0.46 | (-0.50, -0.41) | -0.54 | (-0.58, -0.49) |
| Race/Ethnicity, Hispanic | -0.17 | (-0.21, -0.13) | -0.19 | (-0.25, -0.13) | -0.15 | (-0.20, -0.11) | -0.17 | (-0.22, -0.13) |
| Race/Ethnicity, Non-Hispanic Black | -0.47 | (-0.51, -0.43) | -0.47 | (-0.51, -0.42) | -0.47 | (-0.52, -0.43) | -0.5 | (-0.55, -0.45) |
| Race/Ethnicity, Non-Hispanic Other | -0.3 | (-0.35, -0.24) | -0.3 | (-0.36, -0.24) | -0.31 | (-0.37, -0.26) | -0.32 | (-0.37, -0.27) |
| BMI, Overweight | -0.01 | (-0.05, 0.02) | -0.01 | (-0.06, 0.04) | -0.04 | (-0.08, 0.00) | -0.03 | (-0.07, 0.01) |
| BMI, Obese | -0.11 | (-0.14, -0.07) | -0.12 | (-0.17, -0.07) | -0.12 | (-0.17, -0.08) | -0.11 | (-0.15, -0.07) |
| Individual income, Less than or equal to $15,000/year | -0.02 | (-0.07, 0.02) | -0.01 | (-0.07, 0.04) | -0.02 | (-0.06, 0.03) | 0 | (-0.06, 0.06) |
| Individual income, $15,001 - $30,000/year | -0.03 | (-0.06, 0.01) | -0.02 | (-0.06, 0.02) | -0.03 | (-0.07, 0.01) | 0 | (-0.04, 0.04) |
| Ever Smoking, Yes | -0.09 | (-0.12, -0.06) | -0.1 | (-0.13, -0.06) | -0.07 | (-0.11, -0.04) | -0.07 | (-0.10, -0.03) |
| Insurance, Uninsured | 0.12 | (0.07, 0.17) | 0.09 | (0.03, 0.15) | 0.1 | (0.05, 0.15) | 0.08 | (0.02, 0.14) |
| Education, Equal or less than high school | -0.05 | (-0.08, -0.01) | -0.04 | (-0.08, 0.01) | -0.03 | (-0.07, 0.01) | -0.05 | (-0.09, -0.01) |

| Table B. Summary of additive effect estimates on FEV1 from linear and quantile regression (25th, 50th, and 75th) models for occupational exposure category. | | | | | | | | |
| --- | --- | --- | --- | --- | --- | --- | --- | --- |
|  | Linear regression | | Quantile regression at 25th percentile | | Quantile regression at 50th percentile | | Quantile regression at 75th percentile | |
| Covariate | Estimates | 95% CI | Estimates | 95% CI | Estimates | 95% CI | Estimates | 95% CI |
| Cohort, 2010-2014 | 0.06 | (0.02, 0.09) | 0.03 | (-0.02, 0.08) | 0.08 | (0.04, 0.12) | 0.05 | (0.01, 0.09) |
| Cohort, 2015-2019 | -0.01 | (-0.05, 0.03) | -0.01 | (-0.06, 0.04) | 0.01 | (-0.03, 0.05) | -0.01 | (-0.05, 0.03) |
| Cohort, 2020-2024 | 0.08 | (-0.01, 0.17) | 0.02 | (-0.04, 0.09) | 0.09 | (-0.01, 0.19) | 0.03 | (-0.06, 0.12) |
| Exposure category, Other | -0.05 | (-0.10, -0.00) | -0.05 | (-0.10, 0.01) | -0.01 | (-0.06, 0.05) | -0.01 | (-0.06, 0.04) |
| Exposure category, Worker | -0.07 | (-0.11, -0.03) | -0.06 | (-0.10, -0.02) | -0.04 | (-0.09, -0.00) | -0.05 | (-0.09, -0.01) |
| Age | -0.03 | (-0.03, -0.03) | -0.03 | (-0.03, -0.03) | -0.03 | (-0.03, -0.03) | -0.03 | (-0.03, -0.03) |
| Height | 3.24 | (3.04, 3.44) | 2.95 | (2.69, 3.20) | 3.2 | (2.98, 3.43) | 3.36 | (3.14, 3.58) |
| Sex, Female | -0.45 | (-0.49, -0.41) | -0.4 | (-0.45, -0.35) | -0.47 | (-0.51, -0.42) | -0.54 | (-0.59, -0.50) |
| Race/Ethnicity, Hispanic | -0.16 | (-0.20, -0.12) | -0.19 | (-0.24, -0.13) | -0.15 | (-0.20, -0.11) | -0.19 | (-0.23, -0.14) |
| Race/Ethnicity, Non-Hispanic Black | -0.46 | (-0.49, -0.42) | -0.46 | (-0.50, -0.41) | -0.46 | (-0.50, -0.41) | -0.49 | (-0.53, -0.44) |
| Race/Ethnicity, Non-Hispanic Other | -0.3 | (-0.36, -0.25) | -0.28 | (-0.33, -0.23) | -0.32 | (-0.38, -0.26) | -0.32 | (-0.38, -0.27) |
| BMI, Overweight | -0.01 | (-0.05, 0.03) | 0 | (-0.05, 0.05) | -0.05 | (-0.09, -0.01) | -0.03 | (-0.07, 0.01) |
| BMI, Obese | -0.1 | (-0.14, -0.06) | -0.11 | (-0.15, -0.06) | -0.12 | (-0.16, -0.08) | -0.1 | (-0.14, -0.06) |
| Individual income, Less than or equal to $15,000/year | -0.03 | (-0.07, 0.02) | -0.02 | (-0.07, 0.04) | -0.02 | (-0.06, 0.03) | 0.02 | (-0.03, 0.06) |
| Individual income, $15,001 - $30,000/year | -0.03 | (-0.07, 0.00) | -0.02 | (-0.06, 0.02) | -0.03 | (-0.07, 0.01) | 0 | (-0.04, 0.04) |
| Ever Smoking, Yes | -0.09 | (-0.12, -0.06) | -0.1 | (-0.14, -0.06) | -0.08 | (-0.11, -0.04) | -0.06 | (-0.09, -0.03) |
| Insurance, Uninsured | 0.12 | (0.07, 0.17) | 0.1 | (0.04, 0.15) | 0.1 | (0.06, 0.15) | 0.09 | (0.02, 0.15) |
| Education, Equal or less than high school | -0.05 | (-0.08, -0.01) | -0.04 | (-0.09, 0.01) | -0.04 | (-0.08, 0.00) | -0.05 | (-0.08, -0.01) |

| Table C. Summary of relative effect estimates on AX from linear and quantile regression (25th, 50th, and 75th) models for dust cloud. | | | | | | | | |
| --- | --- | --- | --- | --- | --- | --- | --- | --- |
|  | Linear regression | | Quantile regression at 25th percentile | | Quantile regression at 50th percentile | | Quantile regression at 75th percentile | |
| Coefficient | Estimates | 95% CI | Estimates | 95% CI | Estimates | 95% CI | Estimates | 95% CI |
| Cohort, 2010-2014 | 11.89 | (4.06, 20.30) | 10.99 | (0.96, 22.02) | 9.3 | (0.16, 19.27) | 13.33 | (1.92, 26.02) |
| Cohort, 2015-2019 | 33.85 | (24.93, 43.39) | 39.86 | (28.16, 52.63) | 31.84 | (21.12, 43.51) | 23.84 | (12.23, 36.66) |
| Cohort, 2020-2024 | 26.2 | (8.44, 46.86) | 45.59 | (18.78, 78.46) | 26.67 | (5.47, 52.12) | 31.95 | (9.12, 59.56) |
| Dust cloud, Yes | 10.09 | (4.62, 15.84) | 8.4 | (1.47, 15.81) | 8.91 | (2.67, 15.52) | 9.2 | (1.33, 17.67) |
| Age | 1.83 | (1.59, 2.06) | 1.54 | (1.25, 1.83) | 1.9 | (1.63, 2.17) | 2.04 | (1.70, 2.38) |
| Sex, Female | 58.01 | (49.95, 66.50) | 69.73 | (58.35, 81.93) | 58.49 | (49.13, 68.44) | 55.14 | (43.70, 67.48) |
| Race/Ethnicity, Hispanic | 52.79 | (42.11, 64.28) | 49.42 | (35.51, 64.76) | 65.53 | (51.38, 81.01) | 55.94 | (39.99, 73.72) |
| Race/Ethnicity, Non-Hispanic Black | 101.55 | (88.46, 115.54) | 110.55 | (94.31, 128.15) | 112.13 | (95.47, 130.21) | 97.89 | (81.18, 116.15) |
| Race/Ethnicity, Non-Hispanic Other | 32.39 | (20.75, 45.16) | 36.75 | (19.63, 56.30) | 44.24 | (32.47, 57.05) | 36.38 | (16.91, 59.09) |
| BMI, Overweight | 32.29 | (23.91, 41.23) | 31.7 | (21.31, 42.98) | 36.27 | (26.58, 46.70) | 38.81 | (26.15, 52.74) |
| BMI, Obese | 114.79 | (101.11, 129.41) | 126.7 | (108.32, 146.71) | 119.64 | (104.12, 136.34) | 122.07 | (102.67, 143.34) |
| Individual income, Less than or equal to $15,000/year | 9.59 | (1.54, 18.28) | 0.74 | (-8.38, 10.77) | 5.73 | (-3.54, 15.88) | 15.5 | (1.61, 31.29) |
| Individual income, $15,001 - $30,000/year | 1.77 | (-4.39, 8.33) | -4.96 | (-12.37, 3.08) | -3.93 | (-10.95, 3.65) | 5.4 | (-3.99, 15.70) |
| Ever Smoking, Yes | 12.79 | (6.97, 18.92) | 14.09 | (6.61, 22.10) | 7.19 | (0.57, 14.24) | 20.24 | (11.17, 30.06) |
| Insurance, Uninsured | -13.29 | (-20.78, -5.09) | -13.39 | (-24.31, -0.89) | -10.82 | (-20.00, -0.59) | -12 | (-23.41, 1.11) |
| Education, Equal or less than high school | 14.66 | (7.59, 22.20) | 19.96 | (10.02, 30.81) | 12.81 | (4.56, 21.71) | 13.56 | (2.09, 26.32) |

| Table D. Summary of relative effect estimates on AX from linear and quantile regression (25th, 50th, and 75th) models for occupational exposure category. | | | | | | | | |
| --- | --- | --- | --- | --- | --- | --- | --- | --- |
|  | Linear regression | | Quantile regression at 25th percentile | | Quantile regression at 50th percentile | | Quantile regression at 75th percentile | |
| Covariate | Estimates | 95% CI | Estimates | 95% CI | Estimates | 95% CI | Estimates | 95% CI |
| Cohort, 2010-2014 | 11.73 | (3.86, 20.20) | 11.94 | (1.85, 23.04) | 9.34 | (0.57, 18.86) | 15.99 | (4.06, 29.29) |
| Cohort, 2015-2019 | 33.02 | (24.09, 42.59) | 37.29 | (26.53, 48.98) | 31.54 | (21.05, 42.95) | 24.34 | (12.48, 37.46) |
| Cohort, 2020-2024 | 24.28 | (6.76, 44.67) | 39.91 | (11.76, 75.15) | 23.76 | (5.31, 45.43) | 34.08 | (10.68, 62.44) |
| Exposure category, Other | 4.96 | (-4.24, 15.05) | 6.99 | (-3.71, 18.87) | 4.02 | (-6.50, 15.72) | 8.58 | (-5.59, 24.87) |
| Exposure category, Worker | 11.55 | (4.40, 19.20) | 13.49 | (4.44, 23.32) | 11.64 | (3.97, 19.87) | 9.59 | (-0.65, 20.89) |
| Age | 1.82 | (1.58, 2.06) | 1.57 | (1.31, 1.84) | 1.89 | (1.63, 2.16) | 2.01 | (1.66, 2.36) |
| Sex, Female | 58.73 | (50.59, 67.30) | 70.53 | (59.77, 82.02) | 58.77 | (49.54, 68.57) | 55.05 | (43.55, 67.46) |
| Race/Ethnicity, Hispanic | 50.71 | (40.09, 62.14) | 47.03 | (33.23, 62.26) | 63.43 | (49.26, 78.94) | 55.14 | (38.31, 74.02) |
| Race/Ethnicity, Non-Hispanic Black | 98.53 | (85.48, 112.51) | 105.33 | (90.08, 121.80) | 107.57 | (91.23, 125.31) | 98.68 | (81.93, 116.98) |
| Race/Ethnicity, Non-Hispanic Other | 33.22 | (21.47, 46.10) | 36.51 | (23.77, 50.55) | 44.57 | (33.50, 56.55) | 34.91 | (16.48, 56.25) |
| BMI, Overweight | 32.2 | (23.82, 41.14) | 30.67 | (21.34, 40.71) | 36.51 | (26.80, 46.95) | 36.48 | (23.62, 50.69) |
| BMI, Obese | 113.16 | (99.51, 127.74) | 120.55 | (103.86, 138.61) | 120.6 | (105.43, 136.89) | 123.12 | (102.41, 145.96) |
| Individual income, Less than or equal to $15,000/year | 10.62 | (2.48, 19.40) | 1.2 | (-7.21, 10.38) | 7.26 | (-1.47, 16.76) | 11.96 | (-1.61, 27.41) |
| Individual income, $15,001 - $30,000/year | 3.39 | (-2.91, 10.10) | -3.42 | (-10.19, 3.85) | -0.65 | (-7.73, 6.97) | 4.89 | (-4.46, 15.16) |
| Ever Smoking, Yes | 12.83 | (7.01, 18.97) | 13.36 | (6.55, 20.62) | 8.32 | (1.76, 15.30) | 21.08 | (11.98, 30.92) |
| Insurance, Uninsured | -13.22 | (-20.73, -5.00) | -14.66 | (-23.56, -4.72) | -9.71 | (-19.41, 1.17) | -11.15 | (-23.23, 2.83) |
| Education, Equal or less than high school | 14.34 | (7.27, 21.88) | 19.25 | (11.08, 28.02) | 9.85 | (1.90, 18.43) | 15.43 | (3.68, 28.50) |
